# Recurrent Gonococcemia Reveiling X-linked Properdin Deficiency: A Novel Case Report

**DOI:** 10.1093/ofid/ofaf223

**Published:** 2025-04-10

**Authors:** Colombe Chedal-Anglay, William Vindrios

**Affiliations:** Department of Infectious Diseases, CHU Henri Mondor, Créteil, France; Department of Infectious Diseases, CHU Henri Mondor, Créteil, France

**Keywords:** disseminated gonococcal infections, gonococcemia, immunocompromised patients, properdin deficiency, sexually transmitted infections

## Abstract

We present a unique case involving a patient who was diagnosed with X-linked properdin deficiency after 2 episodes of disseminated gonococcal infections 1 year apart. Although this deficiency is well-documented for its association with meningococcemia, its correlation with disseminated gonococcal infections (DGI) has not been previously reported. Recurrent DGI cases reported in the literature with identified cause are mostly associated with acquired or congenital complement pathway deficiencies. However, properdin deficiency is rarely screened for during a first episode. Our case not only highlights the clinical presentation that should raise suspicion of DGI but also underscores the importance of investigating the alternative complement pathway in such cases. At a time when gonococcal resistance is increasing, it is essential to consider existing strategies for preventing these infections, including vaccinations.

Gonococcal infections can manifest as severe disseminated cases, known as disseminated gonococcal infections (DGI). Given the increasing rates of gonorrhea [1], especially those linked to cephalosporin-resistant strains [2], it is important to alert clinicians to the manifestations of DGI and their association with complement deficiency. As these infections are sexually transmitted, individuals with DGI are also at risk for other sexually transmitted infections (STIs), necessitating comprehensive screening and preventive measures. A first episode of DGI is not always the subject of a thorough etiological investigation. We present a case of a patient with repeated episodes of DGI, who was diagnosed with properdin deficiency following an extensive immunodeficiency assessment. This X-linked deficiency predisposes individuals to other gram-negative diplococci infections, including invasive meningococcal disease. However, there are no reported cases directly linking it to DGI in the literature, suggesting it might be underdiagnosed. This case underscores the importance of detecting properdin deficiency and outlines appropriate management strategies once identified.

## CASE REPORT

### Context

The patient is a 29-year-old Caucasian male who presented with 2 distinct episodes of DGI, occurring 2 years apart. He engages in sexual intercourse with both men and women and occasionally uses recreational drugs like cocaine.

### Medical History

His medical history includes several STIs, including syphilis, condylomas, and asymptomatic rectal carriage of *Chlamydia trachomatis* (CT) and *Neisseria gonorrhoeae* (NG). He has no history of recurrent infections in childhood and no other health issues.

### Initial and Second Presentations

In 2021, he presented with a papulopustular rash featuring purpuric elements affecting the arms, legs, and trunk, accompanied by wrist tenosynovitis and proctocolitis. Blood culture revealed growth of gram-negative diplococci identified as NG, and a positive rectal polymerase chain reaction confirmed CT infection. The DGI responded to a week of cephalosporins, and CT proctocolitis was successfully treated with a week of doxycycline.

The patient presented again in March 2023 with a rapidly progressing necrotic purpuric lesion on the foot evolving over 48 hours. This was associated with fever, forefoot tenosynovitis, and mandibular angle pain. A rheumatologist found no argument for mandibular or ankle arthritis on ultrasound, so no joint puncture was performed and there was no evidence for arthritis rather than arthralgia. His most recent sexual contact occurred 1 week before admission. Suspected bacteremia prompted initiation of cefotaxime after a positive blood culture once again revealed NG. A biopsy of the purpuric lesion, conducted after antibiotic treatment, yielded a sterile culture. Once again, the patient demonstrated rapid clinical improvement in response to antibiotic therapy. Tests for HIV, syphilis, hepatitis B, and hepatitis C were negative.

### Diagnostic Approach

The second episode of DGI prompted us to investigate the possibility of an underlying immune deficiency. Drawing parallels to meningococcus, we recognized that immune defenses against gram-negative diplococci involve the complement system and gamma globulins [3]. The levels of complement C3 (1.55 [.9–1.8] g/L), C4 (.33 [.1–.4] g/L), and CH50 (>61 [31.6–57.6] U/mL) were normal, and there was no hypogammaglobulinemia on the serum protein electrophoresis.

To go deeper, we explored the alternative complement pathway using the alternative pathway 50 (AP50) assay and investigated for properdin deficiency. A low AP50 level <10% (normal range: 60%–140%) and a properdin antigen level below 1.75 mg/L (normal range: 12–40 mg/L) via immunochemistry confirmed the diagnosis of type I properdin deficiency. Next-generation sequencing further confirmed a rare pathogenic variant in the properdin gene, located on the X chromosome: the critical mutation was found at base 235 in exon 4, where the change of cytosine to thymine had generated a stop codon. Although other mutations in exon 4 [4] have been reported, this one has not.

### Medical Management

The diagnosis of properdin deficiency had significant implications for our patient at the individual level. Initially, we followed the French vaccination guidelines for properdin deficiency, which included the meningococcal B vaccine, ACYW tetravalent meningococcal vaccine, pneumococcal vaccine, and *Haemophilus influenzae* type b vaccine. Given his exposure risk to HIV, we also recommended preexposure prophylaxis [5]. He was too old (>26 years old) to meet the criteria for HPV vaccination.

Since properdin deficiency is X-linked, it was crucial to investigate whether the mutation had affected other male members of the family. Consequently, we advised the patient to encourage family members to attend genetic counseling sessions, facilitating the implementation of preventive vaccination measures if deemed necessary. Even though the disease is X-linked and therefore mainly affects men, sisters (and mother) can acquire both abnormal X-genes if each parent carries an abnormal X-gene that is passed on to progeny.

## DISCUSSION

Our case is the first to report on the risk of DGI in the presence of properdin deficiency.

First, it is crucial not to overlook the diagnosis of DGI. Our patient presented 2 episodes illustrating DGI, characterized by a combination of fever, cutaneous signs (small papular or pustular lesions, few in number, located on the extremities), and joint manifestations (tenosynovitis or arthritis) [6]. While endocarditis, meningitis, and osteomyelitis may also occur, they are exceptional. Invasive gonococcal infections differ from typical genital infection manifestations (urethritis, epididymitis, or cervicitis and salpingitis).

When suggestive clinical signs of DGI are present, blood cultures must be performed. *N gonorrhoeae* is fragile and difficult to grow. Blood or synovial fluid cultures are positive in approximately 50% of cases [7]. Our patient is consistent with clinical studies that report a higher frequency of positive blood cultures in patients with cutaneous involvement and tenosynovitis. On the other hand, patients with purulent effusions are more likely to have positive synovial fluid and negative blood cultures. An explanation is that when arthritis is well established, the bacteria are primarily localized in the joint rather than in the bloodstream. Additionally, the severity of arthritis is likely driven more by an immune complex-mediated inflammatory response than by the direct presence of the bacteria [8].

Additionally, other STIs should be screened for, especially in asymptomatic carriers.

Most patients infected with gonococcus do not develop disseminated and severe forms. The traditionally cited rate of DGI is 0.5%–3% of all cases of gonorrhea [9]. Host and microbial factors both contribute to the risk of developing DGI. Certain characteristics of the gonococcal strain enhance tissue invasiveness, particularly the expression of the PorB1A major outer membrane protein. PorB1A strains exhibit a 20-fold higher invasiveness compared to PorB1B strains. Only the second gonococcal strain was sent to the reference center at the Saint-Louis hospital in Paris, and it showed no virulence factors.

Regarding the host, the main risk factors reported in the literature are linked to acquired or congenital complement deficiencies. Crew et al. [10] reported in 2019 a case series describing 9 cases of *N gonorrhoeae* infections in eculizumab-treated patients worldwide, since the start of pivotal clinical trials in 2004 through 2017. Eculizumab is a humanized monoclonal antibody against terminal complement protein C5 that inhibits terminal complement activation. DGI cases revealing complement fraction deficiencies have also been identified [11–13]. Ellison et al. [14] analyzed complement in 22 patients with their first episode of DGI—1 patient had complete deficiency of C1r and 2 had low CH50. One of these patients had low C4 and the other a low C8 level. Thus, complete or subtotal complement defects may be relatively common in patients with DGI than the general population. However, most cases of DGI have not been associated with complement defects. This may be because CH50 and AP50 tests are not routinely performed after the first episode of DGI, but only in case of recurrent invasive neisserial infections. In our opinion, any DGI should be screened for classical immune deficiency with HIV serology, protein electrophoresis, and complement assay with C3 C4 and CH50. If this initial screening is negative, AP50 and properdin should be assayed.

The lack of screening for complement deficiency may explain why our patient is the first reported case of properdin deficiency. The discovery of properdin was credited to Dr. Pillemer of Cleveland, Ohio, USA, in 1954. He identified this serum protein, primarily produced by leukocytes, as crucial for bacterial defense. Properdin is known for its positive regulation of the alternative complement pathway, facilitating the generation and stabilization of alternative C3 convertases [15]. Extensive in vitro studies underscore its pivotal role in defending against *Neisseria* spp, consistent with clinical observations of heightened susceptibility to meningococcal infections [6]. Agarwal et al. [16] found that physiological forms of properdin do not bind directly to Neisseriae but increase AP-dependent C3 deposition on the bacteria, thereby illustrating the AP convertase-stabilizing function of properdin on Neisseriae. The first documented case of properdin deficiency involved a Swedish family where multiple males experienced severe meningococcal meningitis [17]. Located on the X chromosome, mutations in the properdin gene exclusively affect males in families with the genetic variant [18]. Based on immunochemical and functional analyses, 3 distinct types of properdin deficiency has been identified [19]. Type 1 is the most common and is characterized, as in our patient, by a complete absence of both properdin antigen and function. In type 2, properdin deficiency is partial, whereas type 3 deficiency is marked by normal serum levels afunctionally impaired protein.

Men affected by this mutation are particularly susceptible to infections with *N meningitidis*, especially disseminated and severe forms [20–22]. The risk of meningococcal disease in properdin-deficient individuals has been estimated at approximately 50%, which is 250 times higher than that of the general population [20].

As demonstrated in our clinical case, identifying properdin deficiency prompts the implementation of vaccination measures. In 1998, Fijen et al. showed that individuals deficient in properdin respond effectively to immunization with the tetravalent polysaccharide meningococcal vaccine (A, C, Y, W135), generating antibodies and bactericidal anti-meningococcal activity [23]. Current vaccination guidelines stress the importance of vaccines targeting NM serogroup B, *Streptococcus pneumonia*, and *Haemophilus influenzae* type b.

With rising antibiotic resistance in gonorrhea [24], meningococcal vaccination may offer partial protection. The MeNZB vaccine contains outer membrane vesicle proteins, some of which are shared with *N gonorrhoeae*, thereby inducing cross-protective immunity against both *Neisseria meningitidis* and *N gonorrhoeae* [25]. Similarly, 4CMenB vaccine includes recombinant proteins in addition to outer membrane vesicles. One of these, neisserial heparin binding antigen elicits antibodies that cross-react with the neisserial heparin binding antigen of *N gonorrhoeae* [26].

By contrast, DoxyPEP [27] has proven effective in reducing the incidence of CT and syphilis, may have limited, if any, efficacy versus gonorrhea in men having sex with men [28].

## CONCLUSION

We present the first documented case of properdin deficiency revealed through 2 instances of DGI. Historically, this diagnosis has primarily arisen in the context of meningococcal infections, including some fatal cases. However, investigating this deficiency carries significant implications: it facilitates family screening and the implementation of targeted vaccinations for individual protection. The potential efficacy of meningococcal vaccination in protecting against gonococcus suggests a transitional solution in the absence of an effective gonococcal vaccine. Our patient's experience underscores the importance of not underestimating DGI, which may indicate an underlying deficiency predisposing to more severe and preventable infections. We suggest that any DGI with a negative initial immune deficiency panel should be tested for properdin deficiency.

## KEY MESSAGES

- Although rare disseminated gonococcal infections (DGI) should not be overlooked in the presence of fever, dermatitis, and joint manifestations (tenosynovitis or arthritis).
- Blood cultures will be negative in half of all cases, especially in cases of arthritis.
- Given the associated risk of meningococcal disease, a first episode of DGI should be followed by testing for complement deficiency.

## Notes

***Author contributions.*** W.V. and C.C-A. collected the patient data and participated in the treatment. W.V. and C.C-A. wrote the manuscript and approved the final manuscript.

***Patient consent.*** The patient's written consent was obtained. The design of the work conforms to standards currently applied in France. Ethics approval is not needed for Case Reports according to our local Research Ethics Board (Unité de Recherche Clinique Hôpital Henri Mondor, AP-HP, Créteil, France).

***Availability of data.*** All data generated during this study are available from the corresponding author on reasonable request.

***Financial support.*** No funding was received for this case report.

## References

[1] Rowley J, Hoorn SV, Korenromp E, et al Chlamydia, gonorrhoea, trichomoniasis and syphilis: global prevalence and incidence estimates, 2016. Bull World Health Organ 2019; 97:548–62P. 31384073 10.2471/BLT.18.228486 PMC6653813

[2] Radovanovic M, Kekic D, Jovicevic M, et al Current susceptibility surveillance and distribution of antimicrobial resistance in N. gonorrhoeae within WHO regions. Pathogens. 2022; 11:1230. 36364980 10.3390/pathogens11111230 PMC9697523

[3] Lewis LA, Ram S. Meningococcal disease and the complement system. Virulence 2013; 5:98–126. 24104403 10.4161/viru.26515 PMC3916388

[4] Späth PJ, Sjöholm AG, Fredrikson GN, et al Properdin deficiency in a large Swiss family: identification of a stop codon in the properdin gene, and association of meningococcal disease with lack of the IgG2 allotype marker G2m(n). Clin Exp Immunol 1999; 118:278–84. 10540191 10.1046/j.1365-2249.1999.01056.x PMC1905431

[5] Grant RM, Lama JR, Anderson PL, et al Preexposure chemoprophylaxis for HIV prevention in men who have sex with men. N Engl J Med 2010; 363:2587–99. 21091279 10.1056/NEJMoa1011205 PMC3079639

[6] Lohani S, Nazir S, Tachamo N, Patel N. Disseminated gonococcal infection: an unusual presentation. J Community Hosp Intern Med Perspect 2016; 6:31841. 27406461 10.3402/jchimp.v6.31841 PMC4942509

[7] O'Brien JP, Goldenberg DL, Rice PA. Disseminated gonococcal infection: a prospective analysis of 49 patients and a review of pathophysiology and immune mechanisms. Medicine (Baltimore) 1983; 62:395–406. 6415361

[8] Manicourt DH, Orloff S. Gonococcal arthritis–dermatitis syndrome. Study of serum and synovial fluid immune complex levels. Arthritis Rheumatism 1982; 25:574–8. 7082402 10.1002/art.1780250513

[9] Welch G, Reed GW, Rice PA, Ram S. A meta-analysis to quantify the risk of disseminated gonococcal infection with porin B serotype. Open Forum Infect Dis 2024; 11:ofae389. 39035573 10.1093/ofid/ofae389 PMC11259189

[10] Crew PE, Abara WE, McCulley L, et al Disseminated gonococcal infections in patients receiving eculizumab: a case series. Clin Infect Dis 2019; 1;69:596–600. 10.1093/cid/ciy958 PMC6744347 30418536

[11] Davido B, Dinh A, Lagrange A, et al Chronic gonococcal arthritis with C5 deficiency presenting with brief flare-ups: case study and literature review. Clin Rheumatol 2014; 33:1351–3. 24777471 10.1007/s10067-014-2643-x

[12] Ikeuchi K, Okamoto K, Inoue N, Okugawa S, Moriya K. Chronic disseminated gonococcal infection in a Japanese man with novel C5 gene mutation. J Clin Immunol 2021; 41:691–3. 33403467 10.1007/s10875-020-00959-4

[13] Kageyama M, Hagiya H, Ueda Y, et al Disseminated gonococcal infection in a Japanese man with complement 7 deficiency with compound heterozygous variants: a case report. Medicine (Baltimore) 2021; 100:e25265. 33787610 10.1097/MD.0000000000025265 PMC8021336

[14] Ellison RTI, Curd JG, Kohler PF, Reller LB, Judson FN. Underlying complement deficiency in patients with disseminated gonococcal infection. Sex Transm Dis 1987; 14:201–4. 2830678 10.1097/00007435-198710000-00004

[15] Blatt AZ, Pathan S, Ferreira VP. Properdin: a tightly regulated critical inflammatory modulator. Immunol Rev 2016; 274:172–90. 27782331 10.1111/imr.12466 PMC5096056

[16] Agarwal S, Ferreira VP, Cortes C, Pangburn MK, Rice PA, Ram S. An evaluation of the role of properdin in alternative pathway activation on Neisseria meningitidis and Neisseria gonorrhoeae. J Immunol 2010; 185:507–16. 20530262 10.4049/jimmunol.0903598 PMC2933790

[17] Sjöholm AG, Braconier JH, Söderström C. Properdin deficiency in a family with fulminant meningococcal infections. Clin Exp Immunol 1982; 50:291–7. 7151327 PMC1536695

[18] Coleman MP, Murray JC, Willard HF, et al Genetic and physical mapping around the properdin P gene. Genomics 1991; 11:991–6. 1783405 10.1016/0888-7543(91)90024-9

[19] Sjöholm AG . Inherited complement deficiency states: implications for immunity and immunological disease. APMIS 1990; 98:861–74. 2147105 10.1111/j.1699-0463.1990.tb05008.x

[20] Linton SM, Morgan BP. Properdin deficiency and meningococcal disease—identifying those most at risk. Clin Exp Immunol 1999; 118:189–91. 10540177 10.1046/j.1365-2249.1999.01057.x PMC1905414

[21] Frémeaux-Bacchi V, Le Coustumier A, Blouin J, Kazatchkine MD, Weiss L. [Partial properdin deficiency revealed by a septicemia caused by Neisseria meningitidis]. Presse Med 1995; 24:1305–7. 7501623

[22] Cunliffe NA, Snowden N, Dunbar EM, Haeney MR. Recurrent meningococcal septicaemia and properdin deficiency. J Infect 1995; 31:67–8. 8522838 10.1016/s0163-4453(95)91550-8

[23] Fijen CA, Kuijper EJ, Drogari-Apiranthitou M, Van Leeuwen Y, Daha MR, Dankert J. Protection against meningococcal serogroup ACYW disease in complement-deficient individuals vaccinated with the tetravalent meningococcal capsular polysaccharide vaccine. Clin Exp Immunol 1998; 114:362–9. 9844044 10.1046/j.1365-2249.1998.00738.x PMC1905137

[24] Ison CA, Town K, Obi C, et al Decreased susceptibility to cephalosporins among gonococci: data from the Gonococcal Resistance to Antimicrobials Surveillance Programme (GRASP) in England and Wales, 2007–2011. Lancet Infect Dis 2013; 13:762–8. 23764300 10.1016/S1473-3099(13)70143-9

[25] Petousis-Harris H, Paynter J, Morgan J, et al Effectiveness of a group B outer membrane vesicle meningococcal vaccine against gonorrhoea in New Zealand: a retrospective case-control study. Lancet 2017; 390:1603–10. 28705462 10.1016/S0140-6736(17)31449-6

[26] Abara WE, Bernstein KT, Lewis FMT, et al Effectiveness of a serogroup B outer membrane vesicle meningococcal vaccine against gonorrhoea: a retrospective observational study. Lancet Infect Dis 2022; 22:1021–9. 35427490 10.1016/S1473-3099(21)00812-4 PMC10227473

[27] Luetkemeyer AF, Donnell D, Dombrowski JC, et al Postexposure doxycycline to prevent bacterial sexually transmitted infections. N Engl J Med 2023; 388:1296–306. 37018493 10.1056/NEJMoa2211934 PMC10140182

[28] Sankaran M, Glidden DV, Kohn RP, et al Doxycycline postexposure prophylaxis and sexually transmitted infection trends. JAMA Intern Med 2025; 185:266–72. 39761052 10.1001/jamainternmed.2024.7178 PMC11877200

